# CRISPR/Cas9-based targeting of fluorescent reporters to human iPSCs to isolate atrial and ventricular-specific cardiomyocytes

**DOI:** 10.1038/s41598-021-81860-x

**Published:** 2021-02-04

**Authors:** Orlando Chirikian, William R. Goodyer, Elda Dzilic, Vahid Serpooshan, Jan W. Buikema, Wesley McKeithan, HaoDi Wu, Guang Li, Soah Lee, Markus Merk, Francisco Galdos, Aimee Beck, Alexandre J. S. Ribeiro, Sharon Paige, Mark Mercola, Joseph C. Wu, Beth L. Pruitt, Sean M. Wu

**Affiliations:** 1 Stanford University, Stanford, CA USA; 2 Stanford Cardiovascular Institute, Stanford, CA USA; 3 Stanford University School of Medicine, Stanford, CA USA; 4 Biotechnology Graduate Program, California State University Channel Islands, Camarillo, CA USA; 5 Department of Pediatrics, Division of Cardiology, Stanford, CA USA; 6 Department of Cardiology, Utrecht Regenerative Medicine Center, University Medical Center Utrecht, Utrecht University, 3508 GA Utrecht, The Netherlands; 7 Department of Cardiovascular Surgery, German Heart Center Munich, Technische Universität München, Lazarettstraße 36, 80636 Munich, Germany; 8 Insure (Institute for Translational Cardiac Surgery), Department of Cardiovascular Surgery, German Heart Center, Technische Universität München, Lothstraße 11, 80636 Munich, Germany; 9 Department of Medicine, Division of Cardiovascular Medicine, Stanford University , Stanford, CA 94305 USA; 10 Departments of Bioengineering and of Mechanical Engineering, Stanford University, Stanford, USA; 11 Department of Mechanical Engineering, University California, Santa Barbara, CA USA; 12 Biomolecular, Science, and Engineering, University California, Santa Barbara, CA USA

**Keywords:** Genetic engineering, Differentiation, Induced pluripotent stem cells, Cardiac regeneration, Heart development

## Abstract

Generating cardiomyocytes (CMs) from human induced pluripotent stem cells (hiPSCs) has represented a significant advance in our ability to model cardiac disease. Current differentiation protocols, however, have limited use due to their production of heterogenous cell populations, primarily consisting of ventricular-like CMs. Here we describe the creation of two chamber-specific reporter hiPSC lines by site-directed genomic integration using CRISPR-Cas9 technology. In the MYL2-tdTomato reporter, the red fluorescent tdTomato was inserted upstream of the 3′ untranslated region of the Myosin Light Chain 2 (MYL2) gene in order faithfully label hiPSC-derived ventricular-like CMs while avoiding disruption of endogenous gene expression. Similarly, in the SLN-CFP reporter, Cyan Fluorescent Protein (CFP) was integrated downstream of the coding region of the atrial-specific gene, Sarcolipin (SLN). Purification of tdTomato+ and CFP+ CMs using flow cytometry coupled with transcriptional and functional characterization validated these genetic tools for their use in the isolation of bona fide ventricular-like and atrial-like CMs, respectively. Finally, we successfully generated a double reporter system allowing for the isolation of both ventricular and atrial CM subtypes within a single hiPSC line. These tools provide a platform for chamber-specific hiPSC-derived CM purification and analysis in the context of atrial- or ventricular-specific disease and therapeutic opportunities.

## Introduction

Significant advancements in our ability to direct the differentiation of human induced pluripotent stem cells (iPSCs) into various cell types have led to greater understanding in developmental biology and disease pathogenesis as well as improved our ability to discover new drugs and formulate new cell-based therapies. In particular, cardiac-specific differentiation of iPSCs has made it possible to investigate a wide variety of cardiovascular diseases and delve further into the molecular mechanisms underlying early cardiac development.

While human iPSC-derived cardiomyocytes (hiPSC-CMs) have demonstrated significant promise, current differentiation protocols yield heterogeneous cell populations containing mixtures of atrial, ventricular and nodal cells, limiting their potential for selective analysis^1,2^. Many have attempted to isolate cardiomyocytes on the basis of their morphologic (size) or biologic (cell surface markers) characteristics, yet a prevailing approach has yet to be found^3,4^. Further, others have promoted selective differentiation of chamber-specific cell types using small molecules and/or growth factors, but each of these methods lack the levels of purity and reproducibility that are needed in applications such as disease modeling, drug testing, and cell based therapies^5^. Prior in vivo cardiac developmental studies using mouse models^6,7^ informed our decision to create a reporter system using the chamber-specific genes Myosin Light Chain 2 (MYL-2) and Sarcolipin (SLN) to allow for the isolation of homogeneous populations of putative ventricular and atrial hiPSC-CMs, respectively.

MYL-2, encoding for the regulatory light chain associated with cardiac myosin beta heavy chain, is known to be restricted to the ventricular segment of the murine heart tube at E8.0 during early development and maintained into adulthood^8^. Similarly in humans, MYL-2 expression is specific to the ventricles during development through adulthood^9^. Sarcolipin, encoding for a regulator of sarco(endo)plasmic reticulum calcium-ATPase (SERCA), plays a significant role in calcium cycling in both mouse and human hearts and has been found to be expressed exclusively within the atria^10–12^. While SLN is not specific to the heart and is lowly expressed in other tissues (e.g. esophageal and bladder tissues)^10^, the exploitation of SLN as an atrial-specific reporter was favored given the well-established differentiation protocols of hiPSC into cardiac lineages^1,2^.

Using CRISPR-Cas9 technology, we successfully generated three categories of fluorescent reporter lines including ventricular (MYL-2-tandem dimer Tomato (tdTomato) only), atrial (SLN-cyan fluorescent protein (CFP) only), and a double atrial-ventricular (MYL-2-tdTomato; SLN-CFP) reporter lines by targeting fluorescent reporter constructs to these highly-conserved, chamber-specific genes. These tools not only provide novel insights into the morphologic, functional and biological differences in chamber-specific cardiomyocytes, but also provide an effective method for purifying atrial and ventricular populations from the same hiPSC line for further analysis and high-throughput screening.

## Materials and methods

### Maintenance of IPSC cell lines

The human induced pluripotent stem cell lines 113 and C15 were obtained from the Stanford Cardiovascular Institute (SCVI) Biobank. Characterization of both cell lines were performed and no karyotypic abnormalities were observed (Supplemental Fig. 1A). Both cell lines were propagated on Matrigel® coated plates using feeder-free culture conditions (Essential 8) in standard environments consisting of 5% carbon dioxide at 37 °C. Media was changed daily and cells were passaged when cells they became 80% confluent using EDTA.

**Figure 1:**
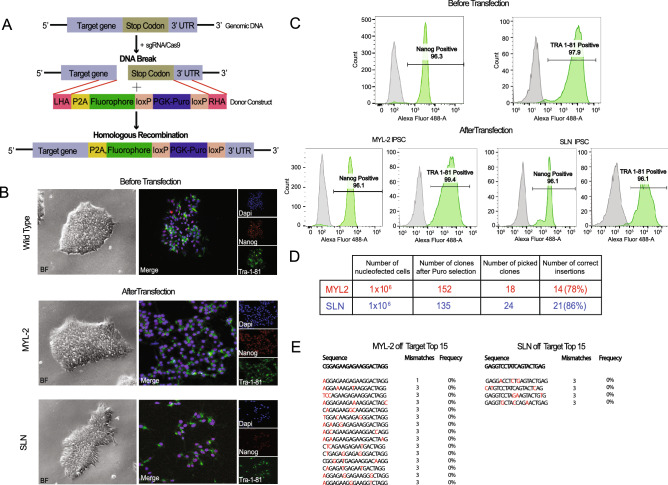
Generation of stable ventricular and atrial reporter lines in human iPSCs. (**A**) Diagram of the CRISPR-Cas9 donor construct design for both the MYL-2/tdTomato and SLN/eCFP. (**B**) Representative images (bright field/Immunofluorescence) of hiPSCs immunostained for pluripotent markers Nanog (red) and Tra-1–81 (green) before and after transfection of donor constructs. (**C**) Representative flow cytometry plots of hiPSCs immunostained for pluripotent markers Nanog and Tra-1–81 before and after transfection. (**D**) A table listing the transfection efficiencies for the development of the MYL-2/tdTomato and SLN/eCFP reporters. (**E**) A list of the top off-target sites (MYL-2 = 15 and SLN = 4) of which had 3 or less base pair mismatches from the targeting guide (red) tested on 10 positive clones respectively.

### Immunostaining FACs analysis and confocal imaging

Cells were fixed either in suspension or adhered to glass slides with 4% PFA. Primary antibodies (1:200) and secondary antibodies (1:500) were diluted in PBS supplemented with 0.1% saponin and 2% Goat Serum. Primary antibodies directed against Nanog (Reprocell,RCAB0002P-F), TRA-1–81 (MilliporeSigma, MAB4381), and cTNT (abcam, MS295P) were incubated for 30 min for cells undergoing flow cytometry analysis or overnight for fixed adherent cells undergoing confocal microscopy imaging. Secondary Alexa 488 or Alexa 564 conjugated antibodies were added for 30 min and counterstained with 4′,6′-diamidino-2-phenylindole (DAPI) or Propidium Iodide (PI) prior to imaging using Zeiss 710 Confocal Microscope.

### RNA extraction and quantitative RT-PCR

RNA extractions were performed using RNeasy Micro Kit (Qiagen) according to manufacturer’s instructions. For each hiPSC or hiPSC-CMs sample, a total of 300 ng of RNA was extracted and purified. Reverse transcription was performed using High Capacity RNA-to-cDNA Kit (Thermo Fisher Scientific) according to manufacturer’s instructions. Quantitative RT-PCR was performed using the HotStart-IT SYBR Green qPCR kit (Affymetrix). Gene expression was normalized to glycerol phosphate dehydrogenase (GAPDH) for all RT-PCR assays. Three biological replicate experiments and two technical replicate experiments were performed on each sample. For time-course experiments the endogenous control values (normalized to GAPDH) on day 0 (for hiPSC samples) or day 30 (for hiPSC-CMs) were used.

### Fluorescence activated cell sorting (FACS)

The BD Flow Cytometry System (Aria II SORP) was utilized for flow cytomety and cell sorting studies. Data analysis was performed using the Flowjo® (TreeStar) software. To optimize the survival of sorted hiPSC-CMs (30–50 days post differentiation), we dissociated and re-suspended hiPSC-CMs in pre-sorting media containing RPMI supplemented with B27, Blebbistatin (1:7000), and Knockout™ Serum Replacement (20%) and placed on ice 20 min prior to sorting. After each sorting, collected cells were centrifuged at 1200 rpm for 5 min to remove sheath fluid and resuspended in replating media containing RPMI supplemented with B27, thiazovivin (1:7000), and Knockout™ serum replacement (10%). Sorted cells were cultured for 6 additional days before further studies.

### Analysis of off-target effects at homologous sites

Evaluation of possible off-target integration was performed by investigating genome sites whose sequences were most similar to the intended cut site and those sites with 4 or less nucleotide mismatches. A total of 10 clones were randomly selected from both cell lines for the assessment of off-target insertions. Genomic DNA was extracted from each clone and targeted genomic PCR for each of the top 10 potential off-target sites was performed.

### Traction force microscopy

Traction Force Microscopy was performed as described previously^13^. Videos of single micropatterned hiPSC-CMs cultured on polyacrylamide hydrogels (10 kPa) with embedded fluorescent microbeads were analyzed to extract traction stresses. For each video, the average displacement of the imaged fluorescent microbeads with time (i.e. velocity) and acceleration of this movement were quantified. Contractile force dipoles were integrated from traction stresses as described previously^13^.

### Automated acquisition of AP kinetics with VF2.1.Cl

Optical imaging of action potential kinetics was conducted as previously described^14^. Briefly, 2 mM VF2.1.Cl was diluted 1:1 in 10% Pluronic F127 and subsequently diluted to a final concentration of 200 nM in Fluorobrite (ThermoFisher) with 4 µg/mL Hoechst 33,258. Cardiomyocytes were imaged at day 30 (15 days after sorting) using the IC200 KIC instrument (Vala Sciences, California, USA) at an acquisition frequency of 100 Hz with excitation wavelength of 485/20 nm and emission filter 525/30 nm using a 0.75 NA 20× Nikon Apo VC objective. Subsequent image analysis was conducted using commercially available Cyteseer software (Vala Sciences) as previously described^14–16^.

### Single Ca^2+ ^transient confocal imaging

Dissociated, MYL2+, and SLN+ hiPSC-CMs were reseeded in Matrigel-coated 384-well glass bottom multi-well plates and were recovered for 3 days. For Ca^2+^ imaging, cells were loaded with Fluo-4 using the Fluo-4 Direct™ Calcium Assay Kit (Thermo Fisher) for 10 min at 37 °C. Cells were then washed with Tyrode’s solution (140 mM NaCl, 5.4 mM KCl, 1 mM MgCl_2_, 10 mM glucose, 1.8 mM CaCl_2_, and 10 mM HEPES, pH adjusted to 7.35 ~ 7.4 with 1 M NaOH at 25 °C) afterwards. Ca^2+^ imaging was recorded with a Zeiss LSM 510Meta confocal microscope (Carl Zeiss AG). Spontaneous Ca^2+^ transients were obtained in a single-cell line scan mode with 40X objective (Plan Apochromat, 0.95 NA), and were analyzed using customized Interactive Digital Language (IDL) script.

### Statistics

Data are presented as mean ± standard error of the mean (SEM) and mean ± standard deviation (SD) when appropriate. Statistical significance was determined by an unpaired t-test. *P* < *0.05* is designated with (*), *P* < *0.005* is designated with (**), *P* < *0.0005* or smaller is designated with (***).

## Results

### CRISPR-Cas9-mediated gene targeting to successfully generate fluorescent reporter lines

To generate chamber-specific fluorescent hiPSC reporter lines, single guide RNAs (sgRNAs) were designed to target genomic DNA sequence immediately upstream of the stop codon for both MYL-2 and SLN respectively (Fig. 1A, Supplemental Fig. 1B). The donor plasmid was constructed using the P2A self-cleaving peptide-based multi-gene expression system allowing for the co-expression of the target genes and their corresponding fluorophores based on endogenous promoter activity without disruption to their biological function. Following transfection of the donor construct into hiPSC lines (C15 and 113) from healthy individuals, puromycin selection was initiated 48 h following transfection. At the end of puromycin selection, 158 MYL-2-tdTomato and 172 SLN-CFP targeted hiPSC clones remained. Successful targeting was confirmed by genome sequencing (Supplemental Fig. 1C). Subsequently, the puromycin resistance cassette was excised by transfection of Cre recombinase expressing plasmids. Pluripotency of the targeted hiPSCs was confirmed before and after transfection using immunofluorescence and flow cytometry analysis for the pluripotency markers such as Nanog and Tra 1–81 (Fig. 1B,C). The efficiency of correct donor construct insertion from MYL-2 and SLN clones was 78% and 86% respectively (Fig. 1D). Sequencing analysis of the PCR product showed no evidence of off target insertions (Fig. 1E).

### Cardiomyocyte directed differentiation validates two distinct populations within the cardiac-lineage

**Figure 2:**
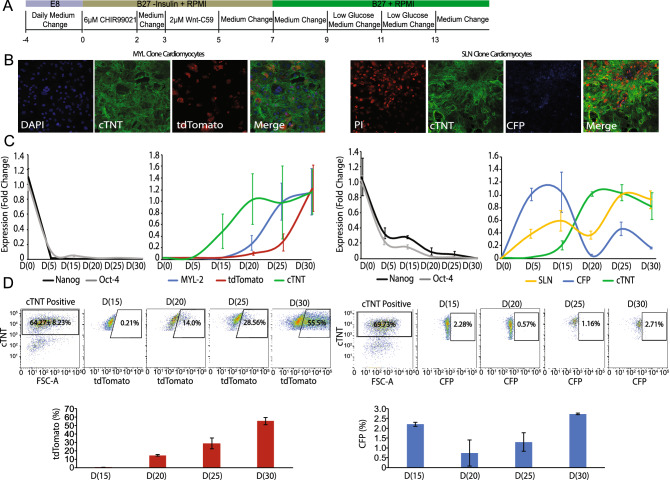
Cardiomyocyte directed differentiation of human iPSC reporter lines. (**A**) Schematic of the cardiomyocyte differentiation protocol^2^. (**B**) Representative Immunofluorescence stainings performed on day 30 cardiomyocytes for both the MYL-2/tdTomato and SLN/eCFP reporters (left to right: nuclear stain (Blue/Red), cTNT (green), fluorescence from reporter (tdTomato, eCFP), and merge). (**C**) Real-time PCR of MYL-2 and SLN reporters over a 30 day duration (increments of 5 days) for pluripotent markers (Nanog and Oct-4), cardiac specific genes (cTNT, MYL-2 and SLN), and respective fluorophores (tdTomato and eCFP) (n = 6). (**D**) Representative flow cytometry plots and corresponding bar graphs from day 15 to day 30 (increments of 5 days) cardiomyocytes depicting the tdTomato(+) and eCFP(+) population (%) within the cTNT(+) population respectively (n = 3).

Once undifferentiated human iPSCs obtained 80% confluency, cardiac differentiation was induced using the directed cardiomyocyte B27 differentiation protocol (Fig. 2A)^2^. Immunofluorescence using anti-cardiac Troponin T (cTNT) was performed on cells obtained 30 days from the start of differentiation, confirming successful differentiation of hiPSCs into cardiac-specific cell lineages (Fig. 2B). In the MYL-2-tdTomato line, robust tdTomato signal was detected in the majority of cTNT + cells. Within the SLN-CFP line, a faint but definitive CFP signal was detectable (Fig. 2B). TdTomato and CFP fluorescence signals were first detected around day 16 and 12 of differentiation, respectively. RNA from days 0, 5, 15, 20, 25, and 30 was obtained for quantitative RT-PCR analysis (Fig. 2C). As expected, the expression of the pluripotency genes Nanog and Oct-4 decreased following initiation of differentiation. Conversely, an increase in cardiac-specific genes including cTNT, MYL-2 and SLN accompanied cardiac differentiation in both reporter lines.

To quantify the amount of tdTomato+ and CFP+ cardiomyocytes within the heterogeneous population of hiPSC-CMs, flow cytometry analysis was performed (Fig. 2D). Within the live cardiomyocyte (i.e. cTNT+) population, tdTomato+ cells gradually increased over time from 0.21 ± 0.02% at day 15 to 55.5 ± 4.24% at day 30. For expression CFP in SLN+ atrial hiPSC-CMs and consistent with prior reports of low abundance of atrial CM differentiation when using the Wnt modulation protocol, the CFP+ population was significantly smaller^2,17^. The percent CFP positive cells transiently decreased from 2.28 ± 0.10% at day 15 to 0.57 ± 0.66% at day 20, with a recovery in percentage of CFP+ cells by day 30 (Fig. 2D).

### Characterization of MYL-2-tdTomato+ cells reveal bona fide ventricular cardiomyocytes

**Figure 3:**
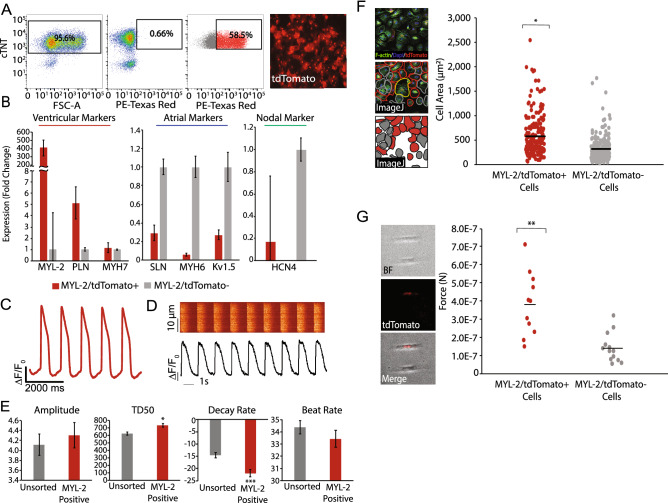
Characterization of the ventricular human iPSC reporter line. (**A**) Representative FACS plots from day 30 cardiomyocytes, depicting the percentage of cells that are cTNT(+), control wild-type cells tdTomato(−), and MYL-2/tdTomato(+) within the cTNT(+) population after sorting. Representative image (right) of sorted MYL-2/tdTomato(+) cells 5 days after sort. (**B**) Real-time PCR of sorted MYL-2/tdTomato (+) cells (day 45) normalized to GAPDH (ΔCT) and tdTomato(−)(ΔΔCT) population (n = 5). (**C**) Representative action potential tracing of MYL-2/tdTomato(+) cells (day 30) using voltage activated fluorescent dye (n = 8). (**D**) Representative calcium tracing and quantification of amplitude, TD50, decay rate, and beat rate of MYL-2/tdTomato(+) cells (day 30) (n = 3). (**E**) Size quantification of day 30 cardiomyocytes, stained for F-Actin (green), Dapi (blue), and fluorescence from MYL-2/tdTomato+ (red) (n = 100). (**F**) Single cell force generation study using traction force microscopy. Images depicting the micropatterned cells (n = 12).

To examine the fidelity of the MYL-2-tdTomato reporter line, we next performed in depth transcriptional and functional characterization of the FACS-purified tdTomato+ cell population (Fig. 3A). By gene expression analysis using RT-PCR, tdTomato+ cells were confirmed to be cardiomyocytes by the presence of cTNT expression (Supplemental Fig. 1D) and further showed an enrichment in ventricular genes (MYL-2, PLN, MYH7) (Fig. 3B). Conversely, there was an expected reduction in the expression of atrial (SLN, MYH6, Kv1.5) and nodal (HCN4) genes within the tdTomato+ population as compared to the tdTomato-population (Fig. 3B).

Next, using a voltage-sensitive dye, we assessed the electrophysiological properties of the tdTomato+ cardiomyocyte population. Consistent with a ventricular CM phenotype, tdTomato+ cells displayed distinct ventricular-like action potentials, including characteristic plateau phases (Phase 2) and accelerated repolarizations (Phase 3) (Fig. 3C)^18^. Using calcium-sensitive dye, we also show that the tdTomato + cardiomyocytes exhibit ventricular-like morphology in its calcium handling properties (Fig. 3D). Specifically, the transient duration (TD50) and calcium recycling (Decay Rate) of tdTomato+ cells were significantly increased as compared to the unsorted population (Fig. 3E)^19^.

### Morphological and contractile characteristics differ in MYL-2-tdTomato + hiPSC-CMs compared to MYL-2-tdTomato-hiPSC-CMs

To further characterize tdTomato+ hiPSC-derived ventricular cardiomyocytes, we assessed the differences in size and contractile force generation between the tdTomato+ and tdTomato− hiPSC-CMs. tdTomato+ and tdTomato− hiPSC-CMs were first stained for F (filamentous)-Actin to demarcate cell boundaries and cell area (µm^2^). From ~ 100 cells analyzed, we found a significant increase in cell area within tdTomato+ hiPSC-CMs compared with tdTomato− hiPSC-CMs (Fig. 3F). Furthermore, using single cell traction force microscopy (TFM), we identified greater contractile force generated in tdTomato+ hiPSC-CMs as compare to tdTomato− hiPSC-CMs (Fig. 3G). These results confirm the ventricular-specific labeling of hiPSC-CM by the expression tdTomato from the MYL2 loci.

### Characterization of SLN-CFP + cells is consistent with atrial cardiomyocytes

**Figure 4:**
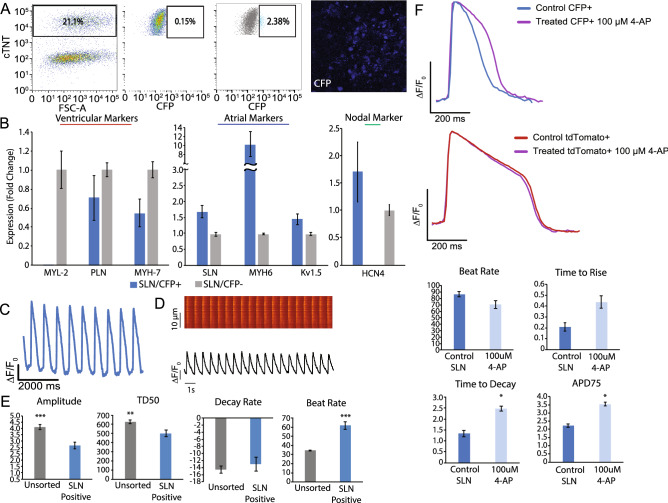
Characterization of the atrial human iPSC reporter line. (**A**) Representative FACS plots from day 30 cardiomyocytes, depicting the percentage of cells that are cTNT(+), control wild-type cells SLN/eCFP(–), and SLN/eCFP(+), within the cTNT(+) population after sorting. Representative image (right) of sorted SLN/eCFP(+) cells 5 days after sort. (**B**) Real-time PCR of sorted cells SLN/eCFP(+) normalized to GAPDH (ΔCT) and SLN/eCFP(−) (ΔΔCT) population (n = 5). (**C**) Representative action potential tracing of SLN/eCFP(+) cells (day 30) using voltage activated fluorescent dye (n = 8). (**D**) Representative calcium tracing and quantification of amplitude, TD50, decay rate, and beat rate of SLN/eCFP(+) cells (day 30) (n = 3). (**E**) Representative action potential tracing depicting APD prolongation in the presence of 100uM 4-AP of sorted SLN/eCFP(+) cells versus control and quantification of beat rate, time to rise, time to decay, and APD75 (n = 8).

To examine the fidelity of SLN-CFP+ expression in atrial hiPSC-CMs, we first isolated CFP+ cells by FACS (Fig. 4A) and profiled their transcriptional signature using RT-PCR. We confirmed the enrichment of cardiomyocyte genes in these CFP+ cells and, consistent with an atrial phenotype, an enrichment for established atrial markers including SLN, MYH6 and Kv1.5 (Fig. 4B, Supplemental Fig. 1D). Reciprocally, we found a reduction in the expression of ventricular genes such as MYL-2, PLN and MYH7. Interestingly, we also observed an increase in nodal gene HCN4 in the CFP+ population as compared to the CFP− population. To further confirm the atrial hiPSC-CM phenotype of CFP+ cells, we used the previously described voltage-sensitive dye to document the electrophysiological properties of the CFP+ population. CFP+ cells exhibit atrial-like action potentials, display increased beat rate, and decreased action potential duration (APD) (Fig. 4C). Furthermore, using a calcium-sensitive dye, we found that the CFP+ hiPSC-CM population displayed atrial-like morphology in its calcium handling properties that is accompanied by a significantly reduced average calcium transient amplitude and TD50 as compared to the unsorted cell population^19^ (Fig. 4D-E).

To further verify that SLN-CFP+ CMs represent a true atrial population, we exposed these cells to 4-aminopyridine (4-AP), thereby inhibiting the atrial-specific ultra-rapid delayed rectifier K^+^ current (I_kur_), mediated by the Kv1.5 channel subunit^20,21^. Consistent with an atrial phenotype, SLN-CFP+ CMs showed a significant increase in action potential duration (APD75) and time to decay as compared to untreated recorded action potentials (Fig. 4F). Notably, exposure of MYL2-tdTomato+ ventricular CMs to 4-AP resulted in no significant action potential changes, consistent with its ventricular phenotype.

### Functional characterization of SLN-CFP+ and MYL2-tdTomato+ cells from a fluorescent double reporter line

**Figure 5:**
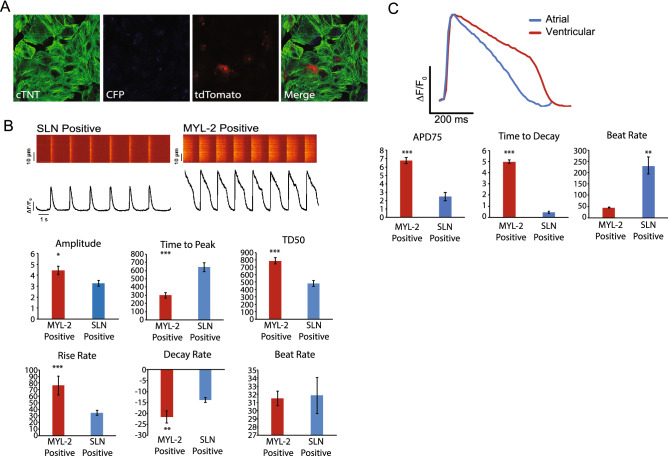
Characterization of the atrial/ventricular human iPSC Double Reporter Line. (**A**) Representative immunofluorescence images of day 30 cardiomyocytes from the double reporter system, MYL-2/tdTomato (red) and SLN/eCFP (cyan), immunostained for cTNT (green). (**B**) Representative calcium tracing and quantification of amplitude, time to peak, TD50, rise rate, decay rate, and beat rate of MYL-2/tdTomato (+) and SLN/eCFP(+) cells (day 30) from the double reporter system. (**C**) Representative action potential tracing of MYL-2/tdTomato (+) and SLN/eCFP(+) cells (day 30) from the double reporter system separately using voltage activated fluorescent dye and quantification of APD75, decay rate, and beat rate of MYL-2/tdTomato (+) and SLN/eCFP(+) cells (day 30) from the double reporter system.

To examine the phenotype of atrial and ventricular hiPSC-CMs from the same hiPSC line following cardiac differentiation, we generated hiPSC lines that contain both MYL2-tdTomato and SLN-CFP reporters in the same line by sequential CRISPR-Cas9-based gene targeting. We selected twelve clones for further characterization and carried out genomic PCR followed by DNA sequencing to confirm correct integration. The integration efficiency for the correct insertion of double reporters using this strategy was 8.33%. As described above, clones with correct reporter integration were assessed for pluripotency markers TRA 1–81 and Nanog by immunostaining and FACS (Supplemental Fig. 2A). We chose one stable double reporter line for in vitro differentiation and assessed the expression of tdTomato and CFP by immunostaining in differentiated cells. As expected, we found consistent expression of tdTomato and CFP in this line (Fig. 5A).

We next characterized this double reporter line functionally by comparing calcium handling in FACS isolated tdTomato+ and CFP+ cardiomyocyte populations. Similar to findings in the hiPSC lines containing single reporters, tdTomato+ hiPSC-CMs from the double reporter line showed a significant increase in calcium transient amplitude, TD50 and decay rate as compared to the CFP+ population, consistent with a ventricular phenotype (Fig. 5B). Reciprocally, CFP+ cells showed a significant increase in calcium transient time to peak, a typical atrial-like calcium handling characteristic^19^.

Next, the action potential (AP) of each isolated CM subtype was directly compared using optical imaging and standard voltage dye analysis. Cardiomyocyte APs for the tdTomato population showed a ventricular-like trace, specifically a prolonged action potential duration (APD75) normalized to beat rate (6.77 ± 0.37 vs 2.49 ± 0.49, respectively. *P* < 0.005) and time to decay (4.99 ± 0.17 vs 0.44 ± 0.09, respectively. *P* < 0.0005) as compared to the CFP populations (Fig. 5C). Consistent with isolation of atrial-like CMs and ventricular-like CMs, we observed a significant increase in beat rate for CFP+ cells as compared to tdTomato+ cells, respectively (229.18 ± 37.64 vs 44.99 ± 1.50, respectively. *P* < 0.005).

## Discussion

The availability of patient-specific human iPSC-CMs have enabled the cardiac field to explore multiple facets of cardiac development, disease and the discovery of novel therapeutics^22^. However, major limitations exist that hinder the use of hiPSC-CMs to their fullest potential. One of the most significant issues remains the generation of mixed atrial, ventricular, and nodal cell populations using current differentiation protocols^17^. The heterogeneity of the cardiac cell populations not only confound biologic findings but prevents our ability to ascertain chamber-specific phenotypes. As a result, our ability capitalize on the power of hiPSCs remains limited.

Here we describe and validate the creation of multiple hiPSC lines using site-directed, fluorescent protein-based reporter system for the isolation of both hiPSC-derived atrial- and ventricular-like CMs. We employed the CRISPR-Cas9 technology to target the insertion of fluorescent reporters downstream of the coding regions of ventricular-specific MYL2 and atrial-specific SLN genes. Our strategy not only allows for the faithful recapitulation of endogenous gene expression but preserves native MYL2 and SLN expression and function. Finally, we demonstrate the ease of gene targeting using these tools by combining both MYL2-tdTomato/SLN-CFP reporter systems within a single hiPSC line allowing for the isolation of both atrial and ventricular CMs from the same cardiac differentiation.

Successful strategies for generating reporter lines depends on multiple criteria including: (1) targeted cell type; (2) reporter gene selection; and (3) the method and site of integration. By using human iPSC lines we have obviated the potential ethical issues and financial limitations surrounding the use of embryonic-derived stem cells^23^. Using the ventricular specific MYL2 gene and atrial-specific SLN gene in both mice and humans alike, we have optimized for the selection of bona fide chamber-specific cardiomyocyte subtypes^9–11,24^. While other groups have adopted transgenic or knock-in strategies, these approaches have two inherent limitations^25,26^. First, transgenic insertion of a reporter risks unfaithful, tissue-specific expression as compared to the endogenous gene by virtue of possibly lacking all promoter and enhancer elements present in the native locus. Second, while strategies that “knock-in” the reporter into the endogenous locus bypass this issue, a significant downside remains the interruption of the coding region of said gene, resulting in haploinsufficiency^26^.

In order to circumvent both of these issues, we have employed CRISPR-Cas9 technology in order to perform site directed integration downstream of the coding region of MYL2 and SLN genes, respectively. By employing the P2A self-cleaving peptide-based multi-gene expression system, we have allowed for the co-expression of the target genes and their corresponding fluorophores based on endogenous promoter activity. Either as single reporter lines or as a combined dual reporter, we have demonstrated the successful isolation of both ventricular- and atrial-like hiPSC-CMs by gene expression and electrophysiologic properties. Additionally, we used 4-AP, an atrial-specific inhibitor of I_kur_ current, to confirm the atrial CM phenotype of SLN-CFP+ cells, highlighting the potential use of this dual reporter system as a platform for high-throughput screening of novel atrial-specific medications. Finally, given the ability to isolate purified populations of both atrial and ventricular CMs within the same differentiation, the dual reporter system also has the potential to provide insight into the morphologic and functional differences in human, chamber-specific cardiomyocytes with unprecedented resolution.

Overall, the tools described here provide a platform for future foundational studies into the morphologic, functional and biological differences in chamber-specific human cardiomyocytes during development. Additionally, the isolation and purification of atrial- and ventricular-specific populations of hiPSC-derived cardiomyocytes holds translational promise for the scientific community by increasing both the accuracy and efficiency of human disease modeling and high-throughput screening for novel therapeutics.

## Supplementary Information

Supplementary figures.
