# Supplementary material for: CRISPR/Cas9-based targeting of fluorescent reporters to human iPSCs to isolate atrial and ventricular-specific cardiomyocytes
Source: Sci Rep. 2021 Feb 4;11:3026. doi: 10.1038/s41598-021-81860-x (PMC7862643; doi:10.1038/s41598-021-81860-x)
Supplement: Supplementary file 1 — Supplementary figures. [file 41598_2021_81860_MOESM1_ESM.pdf]

Supplement 1

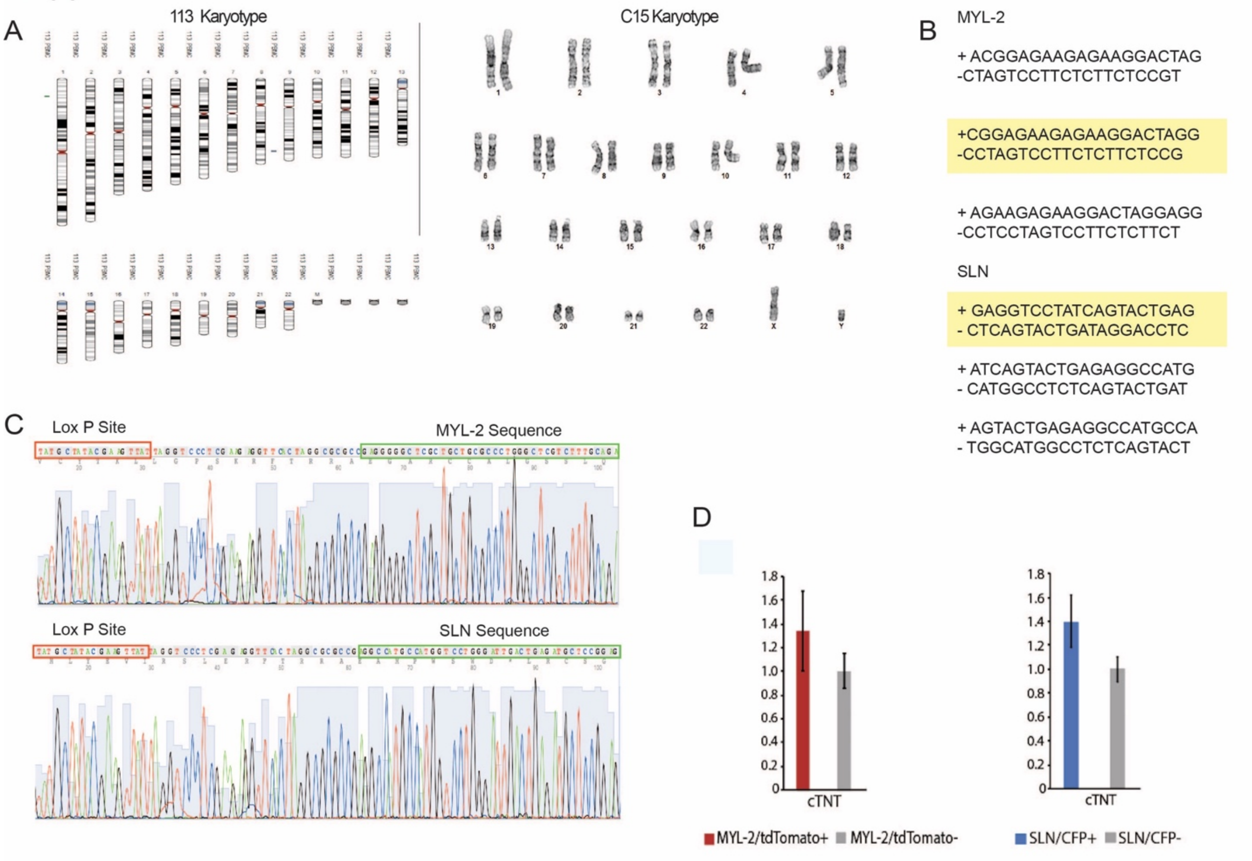

Supplement 2

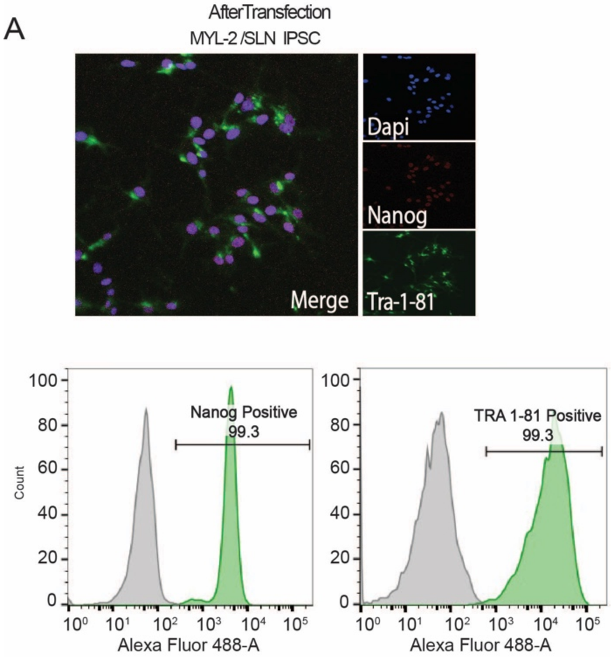

Supplemental 1. A) Karyotype of lines 113 and C15. B) sgRNAs designed for the development of MYL-2/tdTomato and SLN/eCFP reporters (highlighted sgRNA pairs were used for the expirmental development of MYL-2/tdTomato and SLN/eCFP reporters respectively). C) Sanger sequencing data depicting the correct insertion of MYL-2/tdTomato and SLN/eCFP reporter constructs. D) Real-time PCR showing cTNT fold expression levels of MYL-2/tdTomato(+) compared to MYL-2/tdTomato(-) and SLN/eCFP(+) compared to SLN/eCFP(-) respectively.

Supplemental 2. A) Representative images (Immunohistochemistry) of IPSCs stained for pluripotent markers Nanog (red) and Tra-1-81 (green) before and after transfection of donor constructs. Representative Flow Cytometry plots of IPSCs stained for pluripotent markers Nanog and Tra-1-81 before and after transfection. B) Single Cell Real-time PCR of MYL-2/tdTomato (+) cells with varying level of tdTomato signal (high,medium,low).
